# Conservative rehabilitation therapy for respiratory dysfunction due to phrenic nerve sacrifice during resection of massive mediastinal tumor: A case series study

**DOI:** 10.1097/MD.0000000000035117

**Published:** 2023-09-08

**Authors:** Yu Fang, Jun Wu, Maolin Zhang, Yang Yang, Lijun Yao, Lu Liu, Jun Luo, Linjun Li, Cheng Zhang, Zhiming Qin

**Affiliations:** a Department of Thoracic and Cardiovascular Surgery, The First Affiliated Hospital of Chongqing Medical University, Chongqing, China.

**Keywords:** case series study, diaphragmatic dysfunction, massive mediastinal tumor, respiratory dysfunction, ventilator support

## Abstract

**Rationale:**

Cases of respiratory dysfunction due to phrenic nerve sacrifice during resection of massive mediastinal tumor have rarely been studied in detail. Diaphragmatic dysfunction in such cases can lead to potentially fatal respiratory and circulatory disturbances. Therefore, timely diagnosis and intervention are important. Conservative rehabilitation therapy is the first choice for respiratory dysfunction due to diaphragmatic dysfunction.

**Patient concerns, Diagnoses and Interventions:**

We present 3 patients with respiratory dysfunction due to phrenic nerve sacrifice during resection of massive mediastinal tumor. The diagnostic methods and therapeutic procedures for diaphragmatic dysfunction for each patient are described in detail. This study highlights the role of ventilator support combined with physical therapy in the treatment of respiratory dysfunction in such cases. The diagnosis of diaphragmatic dysfunction as well as the risk assessment of phrenic nerve involvement are also discussed. The modalities of ventilator support, including modes and parameters, are listed.

**Outcomes and Lessons:**

This study provides experiences of diagnosis and treatment of respiratory dysfunction due to phrenic nerve sacrifice during resection of massive mediastinal tumor. Timely diagnosis of diaphragmatic dysfunction primarily relies on clinical manifestations and radiography. Conservative rehabilitation therapy can improve or restore diaphragmatic function in majority of patients, and avert or delay the need for surgical intervention. Preoperative assessment of the risk of phrenic nerve involvement is important in such cases.

## 1. Introduction

Phrenic nerve innervates the hemidiaphragm. The phrenic nerves on both sides of thymus are in close proximity to the great vessels and pericardium, and are vulnerable to injury during resection of mediastinal tumors.^[1,2]^ The incidence of phrenic nerve injury during general thoracic surgery is approximately 7%.^[3]^ Phrenic nerve injury can lead to diaphragmatic dysfunction. Severe diaphragmatic dysfunction contributes to restrictive respiratory and circulatory dysfunction,^[4,5]^ necessitating urgent life-saving intervention, such as intubation or ventilator support.

Massive tumors in anterior mediastinum are liable to invade great vessels, pericardium, and the phrenic nerves.^[6]^ Approximately 1 third of patients with advanced stage thymic tumors develop phrenic nerve involvement.^[7]^ Although nerve-sparing surgery followed by adjuvant therapy can achieve similar outcomes compared with phrenic nerve sacrifice, there is a distinct risk of recurrence with the former approach.^[8–10]^ Therefore, extended radical resection, even phrenic nerve sacrifice, is still the priority.^[11]^

Conservative rehabilitation therapy including ventilator support and physical therapy, is the principal therapeutic algorithm for respiratory dysfunction due to phrenic nerve sacrifice.^[12]^ Patients with severe respiratory dysfunction require ventilator support and even intubation.^[13–15]^ Physical therapy (e.g., inspiratory muscle training [IMT]) can partly improve diaphragmatic function, and can complement ventilator support.^[16]^ Surgical approaches (e.g., diaphragmatic plication, phrenic nerve reconstruction) have shown limited benefits in few reports with uncertain outcomes.^[17]^ Herein, we report 3 patients who developed respiratory dysfunction due to phrenic nerve sacrifice during surgical resection of massive mediastinal tumor. This study highlights the role of ventilator support administered in combination with physical therapy in the treatment of respiratory dysfunction due to phrenic nerve sacrifice. In addition, the diagnosis of diaphragmatic dysfunction as well as the risk assessment of phrenic nerve involvement are also discussed. Ethical approval for this study was obtained from the Ethics Committee of the First Affiliated Hospital of Chongqing Medical University. The requirement for written informed consent for this retrospective case report was waived off.

## 2. Case presentation

### 2.1. Case 1

**Figure 1.**
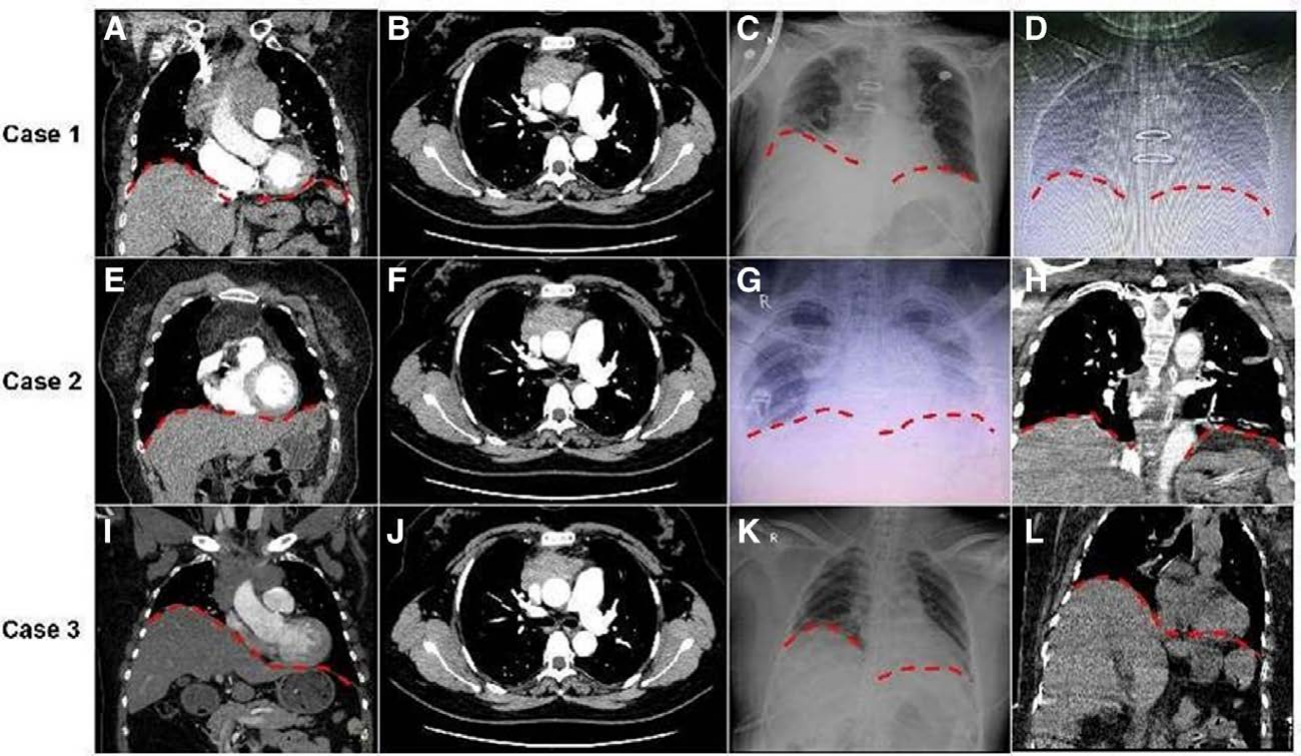
Radiological features of patients with massive mediastinal tumors before and after surgery. (A, E, I) Coronal plane of chest CT before surgery, (B, F, G) Horizontal plane of chest CT before surgery, (C, G, K) Chest X-ray on postoperative day (POD) 2, (D) chest X-ray on POD 20, and (H, L) coronal plane of chest CT on POD 23. Red dotted line indicates diaphragm.

A 70-year-old woman was admitted to our hospital due to chest pain. Chest-enhanced CT showed a giant-sized mass (11.6 × 9.5 × 5.3 cm) with soft tissue density in the anterior mediastinum likely involving the right phrenic nerve (Fig. 1A and B). Histopathological examination of mediastinal puncture specimen indicated thymic carcinoma. Pulmonary function tests showed reduced diffusion function. Open surgery via median sternotomy was performed following general anesthesia and double lumen intubation. Tumorectomy was achieved followed by wedge resection of bilateral upper lobe, superior vena cava plasty, pericardiectomy, left innominate vein resection, and right phrenic nerve resection. The clinical data are listed in Table 1.

**Table 1:** Clinical data of patients with massive mediastinal tumors.

	Case 1	Case 2	Case 3
Symptoms	Chest pain, dyspnea	Dyspnea, chest tightness	Dyspnea, chest pain, cough
Vital signs	Normal	Normal	Normal
BMI (kg/m^2^)	22.4	27.9	29.3
Comorbidity	Local bronchiectasis and atelectasis	Local atelectasis	Hypertension local atelectasis
Pulmonary function	FVC 2.15 (119%), FEV1 1.56 (107%), MVV 50.52 (71%), DLCOS 3.44 (59%)	FVC 2.35 (109%), FEV1 1.93 (119%), MVV 63.58 (104%), DLCOS 5.06 (69%)	FVC 1.69 (66%), FEV1 1.46 (66%), MVV 63.58 (69%), DLCOS 5.06 (69%)
ABG	PaO_2_ 79 mm Hg, PaCO_2_ 34 mm Hg	PaO_2_ 87 mm Hg, PaCO_2_ 35 mm Hg	PaO_2_ 93 mm Hg, PaCO_2_ 37 mm Hg
Electrocardiogram	Abnormal T wave in LV lateral wall	Sinus bradycardia	ST segment change
Echocardiography	Attenuation of LV diastolic function	Mild MVR	Attenuation of LV diastolic function
Blood routine test	WBC count 12.58 × 10^9^/L; NE% 82%	Normal	Normal
Biochemical test	Normal	Normal	Normal
Anti-acetylcholine antibody	Negative	-	Negative
Tumor involvement	SVC, Pericardium, Bilateral upper lobes, LIV, right phrenic nerve	LUL, Pericardium, Left diaphragmatic nerve	SVC, Pericardium, RUL, LIV, right phrenic nerve
Pathology	TC	Liposarcoma	Mixed type thymoma (B2 type and B3 type)

ABG = arterial blood gases, BWI = body weight index, DLCOS = diffusion capacity of carbon dioxide of lung, FEV1 = forced expiratory volume in 1 second, FVC = forced vital capacity, LIV = left innominate vein, LUL = left upper lobe, LV = left ventricular, MVR = mitral valve regurgitation, MVV = maximal ventilatory volume, NE = neutrophil, RUL = right upper lobe, SVC = superior vena cava, TC = thymic carcinoma, WBC = white blood cell.

The patient was transferred to the intensive care unit (ICU) and extubated as normal. On postoperative day (POD) 2, the patient developed progressive sleep disorder and dyspnea. Mild paradoxical thoracoabdominal movement (PTM) was observed in supine position. Arterial blood gas (ABG) analysis showed hypercapnia. Chest x-ray showed elevated right diaphragm (Fig. 1C). The excursion of right and left diaphragm was 6 and 13 mm, respectively, as detected by ultrasound (normal value: 15 ± 4 mm), indicating right diaphragmatic dysfunction. Continuous noninvasive positive pressure ventilation (NIPPV) was suggested based on multi-disciplinary discussion. The modes and parameters of ventilator are listed in Table 2. On POD 12, the continuous NIPPV was changed to intermittent NIPPV. On POD 20, the patient was administered imipenem and cilastatin sodium, because of identification of *Pseudomonas aeruginosa* in sputum culture. On POD 23, the patient was transferred to the general ward with domiciliary NIPPV. The excursion of right and left diaphragm was 9 and 13 mm, respectively, indicating improvement of right diaphragmatic function. Follow-up chest X-ray is shown in Figure 1D. On POD 30, ventilator support was withdrawn and the patient was discharged from the hospital.

**Table 2:** Modes and parameters of ventilator support.

	Dates	ABG	Modes	Parameters
Case 1	POD 2	PaO_2_ 78 mm Hg, PaCO_2_ 71 mm Hg	Continuous NIPPV	IPAP 15 cm H_2_O, EPAP 6 cm H_2_O, f 18/min, FiO_2_ 30%
	POD 12	PaO_2_ 93 mm Hg, PaCO_2_ 36 mm Hg	Intermittent NIPPV	IPAP 15 cm H_2_O, EPAP 6 cm H_2_O, f 18/min, FiO_2_ 30%
	POD 23	PaO_2_ 88 mm Hg, PaCO_2_ 34 mm Hg	Domiciliary NIPPV	IPAP 15 cm H_2_O, EPAP 6 cm H_2_O, f 18/min, oxygen flow 5L/min
	POD 30	PaO_2_ 79 mm Hg, PaCO_2_ 35 mm Hg	Withdrawal of ventilator	-
Case 2	POD 1	PaO_2_ 56 mm Hg, PaCO_2_ 69 mm Hg	SIMV	VT 500 mL, PEEP 0, f 13/min, FiO2 45%
	POD 3	PaO_2_ 137 mm Hg, PaCO_2_ 34 mm Hg	SPONT	VT 500 mL, TiO_2_ 245%, f 16/min
	POD 5	PaO_2_ 97 mm Hg, PaCO_2_ 35 mm Hg	Intermittent NIPPV	IPAP 16 cm H_2_O, EPAP 8 cm H_2_O, f 16/min, FiO_2_ 30%
	POD 11	PaO_2_ 91 mm Hg, PaCO_2_ 37mmHg	Withdrawal of ventilator	-
Case 3	POD 1	PaO_2_ 45 mm Hg, PaCO_2_ 37 mm Hg	v-SIMV	VT 500 mL, PEEP 5 cm H_2_O, f 15/min, FiO2 60%
	POD 9	PaO_2_ 78 mm Hg, PaCO_2_ 38 mm Hg	p-SIMV	PS 25 cm H_2_O, PEEP 5 cm H_2_O, f 13/min, FiO_2_ 45%
			CPAP/PSV	PS 25 cm H_2_O, PEEP 5 cm H_2_O, FiO_2_ 45%
	POD 16	PaO_2_ 72 mm Hg, PaCO_2_ 35 mm Hg	SIPPV	PEEP 4 cm H_2_O, f 22/min, FiO_2_ 35%

ABG = arterial blood gases, CPAP = continuous positive airway pressure, EPAP = expiratory positive airway pressure, IPAP = inspiratory positive airway pressure, NIPPV = noninvasive positive pressure ventilation, PEEP = Positive end-expiratory pressure, POD = postoperative day, PS = pressure support, PSV = pressure support ventilation, SIMV = synchronized intermittent mandatory ventilation, SIPPV = synchronized intermittent positive pressure ventilation, SPONT = spontaneous, VT = tidal volume.

### 2.2. Case 2

A 64-year-old woman was admitted to our hospital because of dyspnea. Six years ago, she had undergone surgery for mediastinal liposarcoma. Chest-enhanced CT showed a massive mass (11 × 6 × 5 cm) with predominant fat density in the anterior mediastinum. The lesion was suspected to have involved the left phrenic nerve (Fig. 1E and F). Pulmonary function tests showed reduced diffusion function. Upon general anesthesia and double lumen intubation, tumorectomy was performed via median sternotomy, which was followed by wedge resection of left upper lobe, pericardiectomy and left phrenic nerve resection. The clinical data are listed in Table 1. The final histopathological diagnosis was recurrent liposarcoma.

The patient was transferred to the ICU and extubated as usual. On POD 1, the patient developed acute-onset dyspnea with mild PTM. ABG showed hypoxemia with hypercapnia. Chest X-ray showed elevation of left diaphragm (Fig. 1G). The excursion of right and left diaphragm was 13 and 6 mm, respectively, indicating left diaphragmatic dysfunction. Continuous NIPPV failed to relieve respiratory dysfunction. Therefore, nasotracheal intubation and mechanical ventilation synchronized intermittent mandatory ventilation (SIMV-mode) were performed immediately. On POD 3, SIMV-mode was switched to SPONT-mode owing to alleviation of manifestations. Physical therapists were invited to perform bedside IMT. On POD 5, the tracheal cannula was removed and intermittent NIPPV was applied. On POD 11, the patient was transferred to the general ward with high flow oxygen inhalation (5 L/minutes). The excursion of right and left diaphragm was 14 and 9 mm, respectively, demonstrating improvement of left diaphragmatic function. The follow-up chest CT is shown in Figure 1H. On POD 20, the patient was discharged from the hospital.

### 2.3. Case 3

A 40-year-old woman was admitted to our hospital with chief complaint of dyspnea. Three months ago, she had undergone resection of pituitary tumor. Chest-enhanced CT showed a massive mass (9 × 8 × 6 cm) with soft tissue density in anterior mediastinum with likely invasion of the right phrenic nerve. Right diaphragmatic elevation was noted (Fig. 1I and J). Histopathological examination of fine needle aspiration biopsy indicated type B2 thymoma. Pulmonary function showed both reduced ventilatory and diffusion function. Upon general anesthesia and double lumen intubation, extended radical surgery was performed via median sternotomy based on the involved tissues. Tumorectomy was followed by wedge resection of right upper lobe, superior vena cava plasty, pericardiectomy, left innominate vein, and right phrenic nerve resection. The left phrenic nerve was spared and protected. The other clinical data are listed in Table 1. The histopathological diagnosis after surgery was mixed type thymoma (B2 type and B3 type).

The patient was transferred to the ICU after surgery and extubated as per routine. Thirty minutes after extubation, the patient developed acute sweating and orthopnea with tachycardia (160–180/minutes) and hypotension (50–60/20–30 mm Hg). ABG indicated severe hypoxemic respiratory failure. Severe PTM was noted. Nasotracheal intubation and mechanical ventilation (v-SIMV-mode) were applied urgently, while vasopressors were administered for circulatory maintenance. Meropenem was also administered due to elevated white blood cell count (30 × 10^9^/L) and neutrophil % (90%). The excursion of right and left diaphragm was 6 and 5 mm, respectively, indicating bilateral diaphragmatic dysfunction. On POD 9, the excursion of right and left diaphragm was 8 and 9 mm, indicating improvement of bilateral diaphragmatic function. After multi-disciplinary discussion, mechanical ventilator support with p-SIMV-mode alternating with continuous positive airway pressure/pressure support ventilation-mode was administered. Physical therapists were also invited to perform IMT at bedside. On POD 10, teicoplanin and tigecycline were added due to identification of *Klebsiella pneumoniae* in sputum culture. On POD 16, alternating mode of ventilator was switched to synchronized intermittent positive pressure ventilation owing to alleviated manifestations. The excursion of right and left diaphragm was 9 and 10 mm, indicating further alleviation of bilateral diaphragmatic dysfunction. Follow-up chest CT is shown in Figure 1L. Unfortunately, the patient developed severe sepsis on POD 23 and died of septic shock.

## 3. Discussion

**Figure 2.**
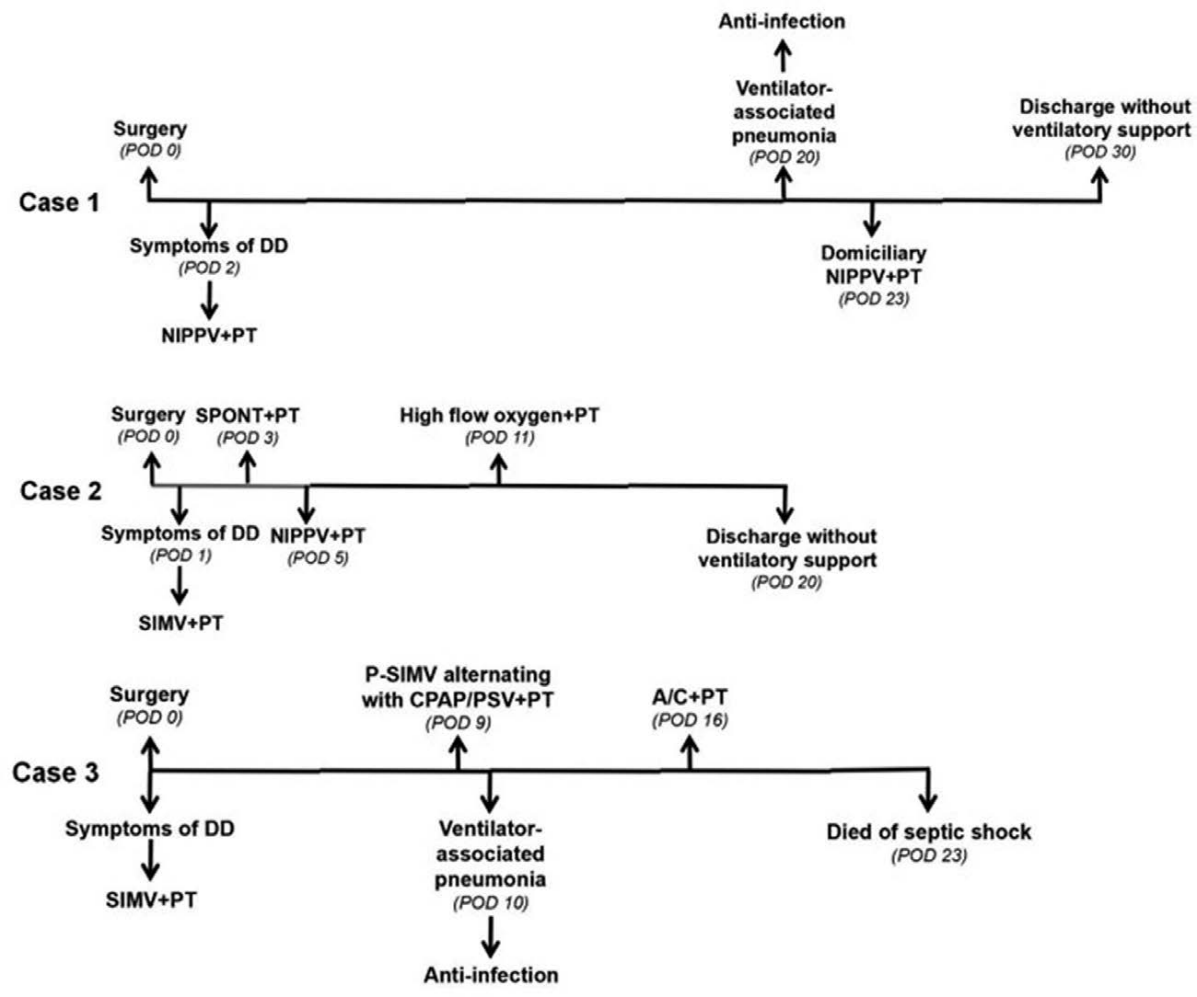
The flow of therapeutic procedures for patients with respiratory dysfunction due to phrenic nerve sacrifice.

In this report, we describe 3 cases of respiratory dysfunction due to sacrifice of phrenic nerve during resection of massive mediastinal tumor. We focus on the therapeutic procedures for respiratory dysfunction caused by diaphragmatic dysfunction (Fig. 2). This study provided experiences on conservative rehabilitation therapy for respiratory dysfunction due to phrenic nerve sacrifice.

The diaphragm serves as a principle inspiratory pump and is innervated by the phrenic nerve.^[18]^ Phrenic nerve injury contributes to diaphragmatic dysfunction. Anterior mediastinal mass, especially those with large size or showing invasive growth, are prone to involve phrenic nerve.^[19]^ Nearly 1 third of all advanced thymomas involve the phrenic nerve.^[20]^ To achieve R0 resection and better prognosis, the involved phrenic nerve had to be sacrificed. However, phrenic nerve palsy may also occur in cases in which the lesion does not invade but is only adjacent to the phrenic nerve.^[21]^ This might be due to stretching, crushing or demyelination of the nerve during surgery, just like the case 3 in the present report.^[22]^ Therefore, preoperative assessment of the risk of phrenic nerve injury is a key imperative. Manifestations and radiography are key indicators for assessment.

Postoperative respiratory dysfunction due to diaphragmatic dysfunction should be diagnosed instantly. Most patients with unilateral diaphragmatic dysfunction are asymptomatic.^[23]^ Associated factors, such as advanced age, obesity, past history of thoracotomy, and underling pulmonary diseases may induce or aggravate symptoms.^[24]^ In this study, patients with unilateral diaphragmatic dysfunction developed severe respiratory symptoms partly due to the synergistic effects of the associated aggravating factors. Unilateral diaphragmatic dysfunction may also contribute to chronic hypercapnic respiratory failure because of mild or moderate hypoventilation,^[25]^ while bilateral diaphragmatic dysfunction can cause acute type I respiratory failure due to severe hypoventilation.^[26,27]^ PTM is attributable to the passive behavior of diaphragm caused by the compensation of accessory inspiratory muscles, and always appears in bilateral diaphragmatic dysfunction.^[28,29]^ In case 3, the patient with bilateral diaphragmatic dysfunction also manifested tachycardia and hypotension in addition to respiratory symptoms, indicating the impairment of cardiac pump function.

Chest X-ray is a simple investigation for evaluating diaphragmatic function.^[30]^ All 3 cases in this study showed postoperative unilateral or bilateral diaphragmatic elevation in chest X-ray. Chest X-ray has been shown to have a high sensitivity (90%) but low specificity (44%) for detecting unilateral diaphragmatic dysfunction.^[30]^ while it is not a reliable method for detection of bilateral diaphragmatic dysfunction. Therefore, more reliable tests are required for diagnosis. Ultrasound is a simple, effective, and convenient investigation for assessment of diaphragmatic dysfunction.^[31,32]^ Ultrasound was shown to have high sensitivity (93%) and specificity (100%) for diagnosing diaphragmatic dysfunction.^[33,34]^ Moreover, it has also been shown to be an effective tool for real-time monitoring during treatment.^[35]^ Other methods for assessment of the function of diaphragm or phrenic nerve include fluoroscopy, phrenic nerve stimulation and electromyography. However, these methods are invasive or are merely limited to experienced institutions.^[26,36,37]^

Once respiratory dysfunction due to diaphragmatic dysfunction is confirmed, specific intervention should be considered. The treatment strategy for diaphragmatic dysfunction predominantly relies on manifestations and ABG analysis. Asymptomatic individuals with unilateral diaphragmatic dysfunction require no intervention. Patients with minimal symptoms (e.g., mild dyspnea, sleep disorders) can benefit from simple measures, such as avoidance of lying on the symptomatic side or in supine position.^[18]^ For patients with chronic hypercapnic respiratory failure due to the slow-onset of diaphragmatic dysfunction, noninvasive continuous positive airway pressure has been suggested first. In case of lack of improvement of hypoventilation and ABG, NIPPV has been recommended.^[14]^ Patients with hypoxemic respiratory failure due to acute-onset of diaphragmatic dysfunction require intubation and mechanical ventilation. The choice of ventilator mode (e.g., SIMV, synchronized intermittent positive pressure ventilation, SPONT) predominantly depends on the manifestations and ABG analysis, which was shown to be associated with the improvement or recovery of diaphragmatic function and compensation of accessory respiratory muscles.^[13]^ In this study, sequential de-escalation of ventilator modes (from invasive ventilation to noninvasive ventilation, from SIMV to SPONT) was used according to periodical follow-up of diaphragmatic function.

Several other principles should be followed for quick restoration of diaphragmatic function; Comorbid disorders (e.g., infection, myasthenia gravis, heart failure) aggravating respiratory dysfunction during therapy should be treated as priority.^[38,39]^ In this study, patients with ventilator-associated infection were administered corresponding sensitive antibiotics, while those with hypotension and tachycardia upon extubation were instantly administered vasopressor; The benefits of physical therapy are limited. Studies have shown that IMT is only a supplementary means to improve diaphragmatic function, which provides short-term benefits;^[40]^ Tracheotomy is indicated for patients with prolonged intubation or difficulty in airway management. In our patients, tracheotomy was not considered due to possibility of mediastinal infection within the first 4 weeks after sternotomy; Surgical approaches, such as diaphragmatic plication or phrenic nerve reconstruction, are not recommended at the onset of respiratory symptoms, because diaphragmatic dysfunction can improve spontaneously, especially under a rehabilitation plan.^[41,42]^

## 4. Conclusion

This study provided some experiences of diagnosis and treatment of patients with severe respiratory dysfunction due to phrenic nerve sacrifice during resection of massive mediastinal tumor. Timely diagnosis of diaphragmatic dysfunction is crucial and primarily relies on clinical manifestations and radiography. Conservative rehabilitation therapy can improve or restore diaphragmatic function in most cases. In addition, preoperative assessment of the risk of phrenic nerve injury during resection of mediastinal tumors is a key imperative.

## Acknowledgments

We thank Medjaden Inc. for scientific editing of this manuscript.

## Author contributions

**Conceptualization:** Yu Fang, Zhiming Qin.

**Data curation:** Yang Yang, Lijun Yao, Linjun Li, Cheng Zhang.

**Formal analysis:** Jun Wu, Linjun Li.

**Funding acquisition:** Zhiming Qin.

**Investigation:** Maolin Zhang, Yang Yang, Linjun Li.

**Methodology:** Yang Yang, Lijun Yao, Lu Liu.

**Project administration:** Lu Liu, Jun Luo.

**Resources:** Jun Wu, Lu Liu, Jun Luo.

**Supervision:** Yu Fang.

**Validation:** Yu Fang, Maolin Zhang, Cheng Zhang, Zhiming Qin.

**Writing – original draft:** Lijun Yao, Zhiming Qin.

**Writing – review & editing:** Jun Luo, Cheng Zhang, Zhiming Qin.
